# The window period of NEUROGENIN3 during human gestation

**DOI:** 10.4161/19382014.2014.954436

**Published:** 2014-10-31

**Authors:** Rachel J Salisbury, Jennifer Blaylock, Andrew A Berry, Rachel E Jennings, Ronald De Krijger, Karen Piper Hanley, Neil A Hanley

**Affiliations:** 1 Center for Endocrinology and Diabetes; Institute of Human Development; Faculty of Medical & Human Sciences; Manchester Academic Health Sciences Center; University of Manchester; Manchester, UK; 2 Endocrinology Department; Central Manchester University Hospitals NHS Foundation Trust; Manchester, UK; 3 Erasmus MC; University Medical Center; Rotterdam, The Netherlands; 4 Department of Pathology; Reinier de Graaf Hospital; Delft, The Netherlands

**Keywords:** development, endocrine, fetal, human, Neurogenin-3 (NEUROG3), pancreas, sex-determining region Y-box 9 (SOX9)

## Abstract

The basic helix-loop-helix transcription factor, NEUROG3, is critical in causing endocrine commitment from a progenitor cell population in the developing pancreas. In human, NEUROG3 has been detected from 8 weeks post-conception (wpc). However, the profile of its production and when it ceases to be detected is unknown. In this study we have defined the profile of NEUROG3 detection in the developing pancreas to give insight into when NEUROG3-dependent endocrine commitment is possible in the human fetus. Immunohistochemistry allowed counting of cells with positively stained nuclei from 7 wpc through to term. mRNA was also isolated from sections of human fetal pancreas and *NEUROG3* transcription analyzed by quantitative reverse transcription and polymerase chain reaction. NEUROG3 was detected as expected at 8 wpc. The number of NEUROG3-positive cells increased to peak levels between 10 wpc and 14 wpc. It declined at and after 18 wpc such that it was not detected in human fetal pancreas at 35–41 wpc. Analysis of *NEUROG3* transcription corroborated this profile by demonstrating very low levels of transcript at 35–41 wpc, more than 10-fold lower than levels at 12–16 wpc. These data define the appearance, peak and subsequent disappearance of the critical transcription factor, NEUROG3, in human fetal pancreas for the first time. By inference, the window for pancreatic endocrine differentiation via NEUROG3 action opens at 8 wpc and closes between 21 and 35 wpc.

## Abbreviations

NEUROG3: Neurogenin-3
SOX9: Sex-determining region Y-box 9
wpc: weeks post-conception
dpc: dayspost-conception

## Introduction

Understanding β-cell differentiation is central to appreciating how β-cell mass is generated in preparation for post-natal life. This early step is especially pertinent as postnatal β-cell proliferation is highly restricted in human compared to rodent.^1,2^

Studies predominantly in mouse have demonstrated conclusively that the basic helix-loop-helix (bHLH) factor, Neurogenin 3 (NEUROG3, also known as NGN3), is required for endocrine commitment.^3,4^ This occurs in a population of progenitor cells themselves regulated by key transcription factors, such as Sex determining region Y-box 9 (SOX9) and pancreas and duodenal homeobox 1 (PDX1).^3,4^ In mouse development, Neurog3 is detectable in 2 phases from embryonic day (e) 8.5 to e11 and during so-called ‘secondary transition’ from ∼e12 to e18, peaking at e15.^5,6^ In human, there is evidence that the transcription factor is required for endocrine cell differentiation; among several reported cases, a severely inactivating mutation in *NEUROG3* caused permanent neonatal diabetes with hyperglycemia the day after birth and extreme loss of pro-endocrine function in *in vitro* and *in vivo* assays.^7^ In early human embryos there is no first phase of NEUROG3 but the transcription factor becomes progressively detected immediately after the embryonic period at ∼8 weeks post-conception timed with the appearance of the first fetal β-cells.^8^ NEUROG3 has been reported in the human fetal pancreas until mid-gestation.^9-11^ However, the relative profile of its expression, such as when its detection peaks, has been unclear; and it is unknown whether and, if so, when NEUROG3 ceases to be detected during human pre-natal development. Here, we have studied a series of human fetuses to provide more complete knowledge of when NEUROG3-dependent endocrine commitment is possible during human gestation.

## Results

**Figure 1.**
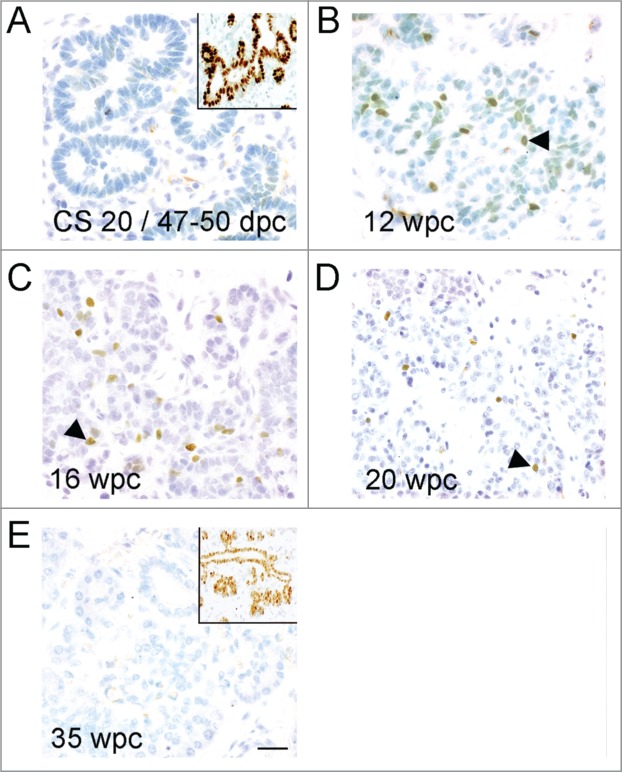
Profile of immunohistochemistry for NEUROG3 during human development. (**A**–**E**) Brightfield images show NEUROG3 in brown counterstained with toluidine blue. Insets (**A**) and (**E**) show brown SOX9 staining in nearby sections from the same fetus. Arrowheads exemplify positively stained cells. Scale bar represents 50 μm in all panels.

**Figure 2.**
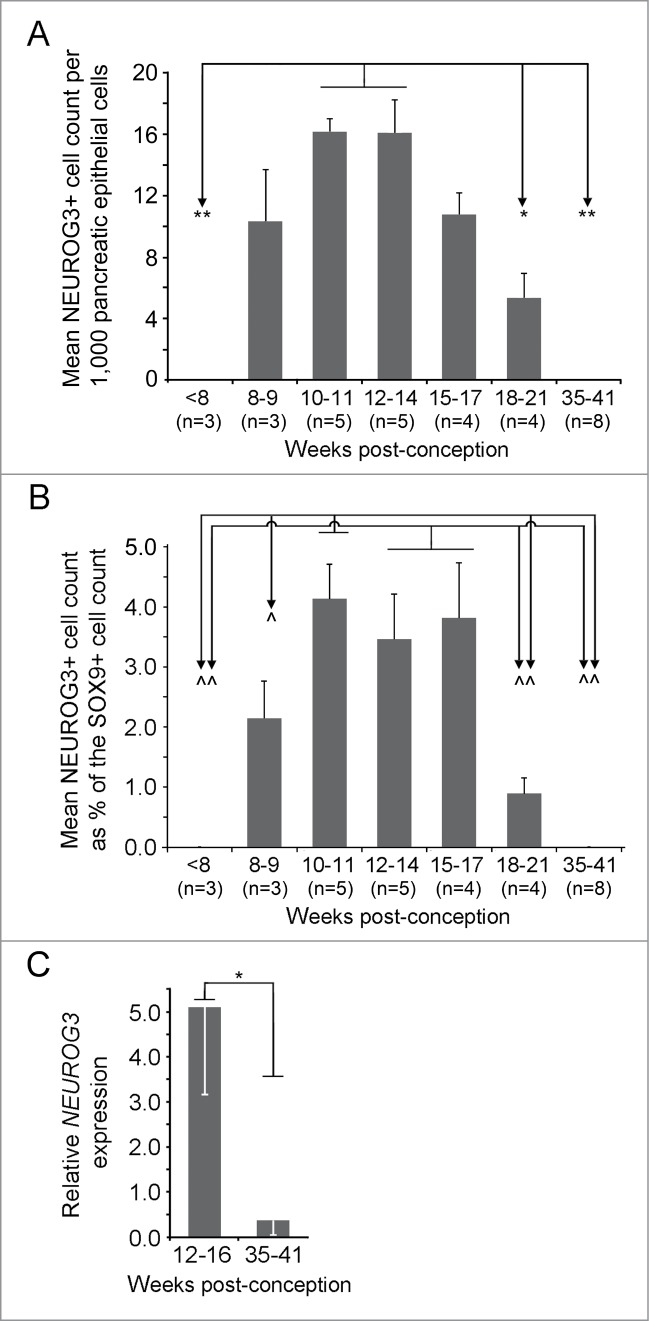
Quantification of NEUROG3 by cell-counting and mRNA analysis during human fetal development. (**A**) and (**B**) The NEUROG3 count was quantified relative to the total pancreatic epithelial cell population (**A**) and the SOX9-positive cell population (**B**). By either approach the NEUROG3 count at 10-11 wpc and at 12–14 wpc were statistically greater than at <8 wpc, 18–21 wpc and 35–41 wpc. When quantified relative to the SOX9-positive cell population, 10-11 wpc, 12–14 wpc and 15–17 wpc emerged as the period of peak NEUROG3 detection. In neither (**A**) nor (**B**), was there a significant difference between counts at 10–11, 12–14 and 15–17 wpc. *P < 0.05, **P < 0.01, ^*P* < 0.005, ^^*P* < 0.0001 by one-way ANOVA with post-hoc Tukey's test. The number of specimens in each group (n) is shown in parentheses. (**C**). Relative *NEUROG3* expression by qRT-PCR demonstrated higher levels in samples from 12–16 wpc than at 35–41 wpc. *P < 0 .01 by 2-tailed unpaired Student's t-test.

We counted cells showing NEUROG3 immunoreactivity in specimens from Carnegie Stage 20 (47–50 dpc) until term. Acinar differentiation has commenced prior to the first detection of NEUROG3 immunoreactivity^8^ and is the cell lineage most responsible for increasing pancreatic size during development. Therefore, we quantified NEUROG3 detection both as a percentage of total pancreatic cells and as a percentage of the SOX9-positive progenitor cell population. NEUROG3 was absent <8 wpc but became progressively more apparent later in the first trimester (**Figs. 1A-B** **and** **2A-B**). Whether compared to either total pancreatic cells or the SOX9-positive cell population, the profile of NEUROG3 detection was very similar (**Fig. 2A-B**). The highest prevalence of NEUROG3-positive cells as a proportion of total epithelial cells occurred between 10 wpc and 14 wpc. When expressed as a proportion of the SOX9-positive population (i.e., excluding differentiated acinar cells^8^), the highest levels of detection extended from 10 wpc until 17 wpc (**Figs. 1C** **and** **2A-B**) when NEUROG3-positive nuclei were ∼3.5–4% of the number of cells stained for SOX9. After this period, NEUROG3 declined such that it was not detected in all 8 of the specimens tested at or after 35 wpc (**Figs. 1D-E** **and** **2A-B**). In all specimens, nuclear immunoreactivity was detected for SOX9 (**Fig. 1A and E** insets) and other transcription factors (data not shown) confirming satisfactory integrity of the specimens. We did not detect NEUROG3 staining in human pancreas either during the first postnatal year or in adulthood (data not shown).

**Figure 3.**
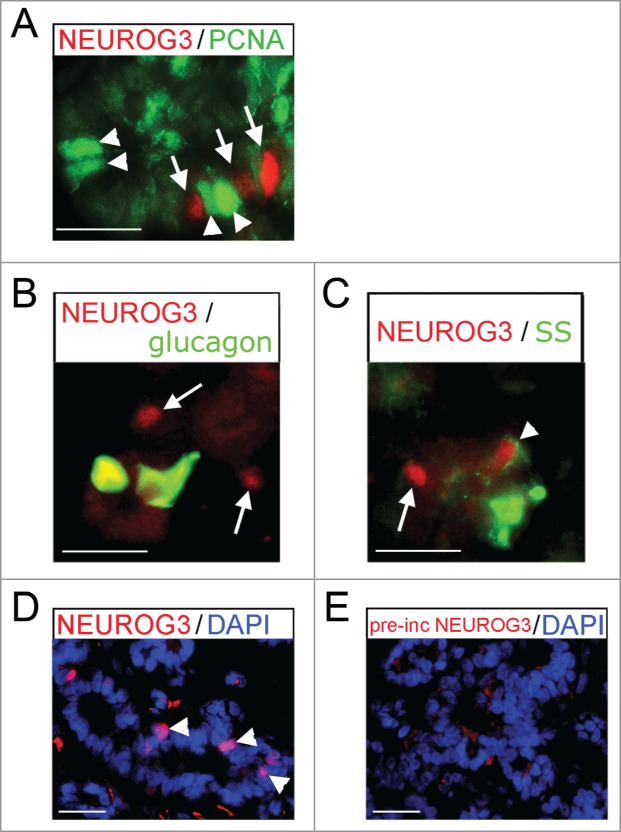
Immunofluoresence for NEUROG3 in human fetal pancreas. (**A**–**E**) Immunofluorescence for NEUROG3 at 14 wpc. (**A**) Arrowheads point to green PCNA-positive cells while arrows point to separate red NEUROG3-positive cells. (**B**) and (**C**) Arrows point to hormone-negative NEUROG3-positive cells. Arrowhead points to a very rare NEUROG3-positive cell with faint somatostatin (SS) staining. (**D**) and (**E**) Arrowheads point to NEUROG3-positive nuclei visible in (**D**) but not (**E**) following pre-incubation with full-length human NEUROG3 protein. Amplification of the red gain with DAPI counterstaining to investigate the loss of nuclear staining has introduced some background cytoplasmic staining. Scale bar represents 50 μm.

We have previously demonstrated that NEUROG3 production in progenitor cells is marked by the exclusion of both PDX1^10^ and SOX9,^8^ and that NEUROG3 could not be detected in insulin-positive cells.^10^ Here, we also showed that cells containing nuclear NEUROG3 were non-proliferative by virtue of absent PCNA staining (**Fig. 3A**) and did not contain glucagon (**Fig. 3B**). Very occasional NEUROG3-positive cells possessed weak staining for somatostatin (arrowhead in **Fig. 3C**). The anti-NEUROG3 antibody was raised against the mouse protein. In addition to the pre-incubation control data shown previously,^10^ here we demonstrate loss of staining following pre-incubation specifically with the in vitro transcribed and translated full length human NEUROG3 protein (**Fig. 3D-E**). Finally, to corroborate the protein data, we performed qRT-PCR. Sample exhaustion precluded exactly mirroring the range of specimens used to detect protein. However, we analyzed *NEUROG3* expression during the window period of its high detection (12–16 wpc) and once we could no longer detect NEUROG3 immunostaining (35–41 wpc) (**Fig. 2C**). Concordant with the protein data there was a >10-fold decline in *NEUROG3* transcripts from 12–16 wpc to very low levels near term when the protein was no longer detected.

## Discussion

Significant work has been reported in recent years on human β-cell numbers, proliferation and apoptosis as integral factors in postnatal β-cell mass.^1,2^ However, due to restricted access to human fetal material, no data exist on whether endocrine differentiation ceases during human development and, if so, when. In mouse pancreas, Neurog3 is required for islet cell differentiation.^3,4^ Studies have also shown Neurog3 to be required for the maintenance of islet cell function in adult mice.^12,13^ An equivalent role in endocrine differentiation seems highly likely in human given the neonatal diabetes and undetectable glucagon when NEUROG3 was severely inactivated.^7^ However, the relative levels and prevalence of NEUROG3 during human gestation has not been examined. In this brief report, we wanted to delineate the profile of NEUROG3 in the developing human pancreas to define the window period when NEUROG3-dependent endocrine commitment is feasible and, specifically, whether this window closes during gestation. We chose to quantify NEUROG3-positive cells in 2 ways, each with its advantages and drawbacks. The most straightforward approach expressed stained cells relative to the total population of pancreatic epithelial cells (**Fig. 2A**). However, as development proceeds beyond embryogenesis this approach includes differentiated acinar cells in the denominator. As an alternative denominator the SOX9-positive population excludes acinar cells by 14 wpc^8^ (**Fig. 2B**). However, at later time points in development not all SOX9-positive cells are likely to be progenitors capable of endocrine differentiation as terminally differentiated adult duct cells also contain SOX9.^10^ By either approach the profile of NEUROG3 was very similar.

Our data at the onset of NEUROG3 detection were very similar to those previously published from our own group and others^8-11^; and are consistent with data from mouse indicating that production of Neurog3 almost entirely marks non-proliferative cells.^3^ NEUROG3 was absent prior to 8 wpc (in Jennings et al, one specimen of the 3 studied prior to 8 wpc contained 2 positive cells^8^). By either counting method, the peak NEUROG3 detection included the period from 10 wpc to 14 wpc. At 15–17 wpc the NEUROG3-positive population remained at approximately 3.5–4% of the SOX9-positive population (**Fig. 2B**) but was slightly lowered when expressed relative to the total pancreatic cell population, possibly due to an increase in acinar cells at the start of the second trimester.^14^ By both counting methods, NEUROG3 then declined implying that the window of NEUROG3-dependent endocrine commitment during human gestation closes between 21 wpc and 35 wpc. Interestingly, a recent study of human fetal pancreas obtained at 7–9 wpc and engrafted into mouse muscle showed NEUROG3 immunoreactivity at 19 weeks post-engraftment but not at the next time point studied, 37 weeks.^15^ While caution is needed in extrapolating from these human xenografts to normal gestation, pooling our data would narrow the end of endocrine commitment to between 26–28 wpc (i.e. 7–9 wpc at transplantation +19 weeks incubation in vivo^15^) and 35 wpc. Our data on *NEUROG3* transcripts were supportive of the cell-counting data: while tissue exhaustion meant that we could not conduct identically timed analyses, *NEUROG3* transcripts were readily detected at or near the peak prevalence of NEUROG3-positive cells but greatly diminished once protein was no longer detected. Previously, we have reported a lack of NEUROG3 in fetal β-cells ^10^ and here show the same for fetal α-cells. However, somatostatin was weakly apparent in very occasional NEUROG3-positive cells perhaps suggestive of very early delta-cell differentiation following the production of NEUROG3. Others have reported NEUROG3 in human adult islets.^12^ We did not detect NEUROG3 at or after 35 wpc prenatally or postnatally. However, the sensitivity of our immunohistochemistry does not preclude cells that were negative here having lower levels of NEUROG3 protein.

In summary, these combined data demonstrate the profile of NEUROG3 detection during human gestation for the first time. Alongside the knowledge that Neurog3 is critical for mouse endocrine commitment^3,4^ and severe mutation causes permanent neonatal diabetes in human,^7^ the combined implication is that NEUROG3-dependent endocrine differentiation in human is maximal at 10–17 wpc. Our data also imply that NEUROG3-dependent endocrine differentiation ceases at some point between 21 and 35 wpc after which further increases in prenatal β-cell mass would presumably be reliant upon the balance of β-cell proliferation versus apoptosis.

## Methods

### Human tissue

Fetal control material was obtained as described previously with informed consent and ethical approval^8,10,16^ or obtained anonymously according to the code established by the Dutch Federation of Medical Scientific Societies (http://www.federa.org) for appropriate secondary use. Thus, specimens were acquired from either social / voluntary termination of pregnancy or from death not related to the pancreas.

### Immunohistochemistry, immunofluorescence and cell counting

Immunohistochemistry and immunofluorescence were performed as described previously^8,16^ on 5 μm sections of pancreas using the primary antibodies and conditions listed in Supplementary **Table 1**. Cell counting data are presented as mean ± standard error of the NEUROG3-positive population either relative to the total pancreatic cell population or as a percentage of the SOX9-positive population. Quantification of positively stained cells for NEUROG3 and SOX9 was from entire serial sections of fetal pancreas at ≥2 different positions from ≥3 separate fetuses within each age group (total n = 32).

### Isolation of RNA, reverse transcription and quantitative PCR

Total RNA was isolated from tissue sections using the Qiagen RNeasy FFPE kit protocol according to the manufacturer's instructions. Reverse transcription (RT) and quantitative PCR (qRT-PCR) were performed as described previously using the ΔΔCT method standardized to 2 housekeeping controls, *GAPDH* and *β-ACTIN*. Primers are listed in Supplementary **Table 2**.

### Statistical analysis

Cell counting across different age groups was compared by one-way ANOVA followed by Tukey's post-hoc test. qRT-PCR between 12–16 wpc and 35–41 wpc was compared by a 2-tailed unpaired Student's t-test.

## Supplemental Materials

Supplemental data for this article can accessed on the publisher's website.
