# Supplementary material for: The window period of NEUROGENIN3 during human gestation
Source: Islets. 2014 Oct 31;6(3):e954436. doi: 10.4161/19382014.2014.954436 (PMC4376053; doi:10.4161/19382014.2014.954436)
Supplement: Supplementary_Tables.docx [file kisl-06-e954436-s001.docx]

**Supplementary Tables**

| **Primary Antibody** | **Raised In** | **Dilution** | **Supplier** |
| --- | --- | --- | --- |
| Monoclonal anti-NEUROG3 | Mouse | 1:1500* | DSHB |
| Polyclonal anti-PCNA | Rabbit | 1:1000 | Abcam |
| Polyclonal anti-SOX9 | Rabbit | 1:5000 | Millipore |

### Supplementary Table 1. Primary antibodies

The antibody developed by O.D. Madsen (anti-NEUROG3) was obtained from the Developmental Studies Hybridoma Bank developed under the auspices of the NICHD and maintained by the University of Iowa, Department of Biology, Iowa City, IA 52242. *Similar results were obtained with dilutions between 1:1000 and 1:2000.

| **Gene** | **Accession no.** | **Forward Primer** | **Reverse Primer** | **Size (bp)** |
| --- | --- | --- | --- | --- |
| *β-ACTIN* | NM_001101.3 | CCAACCGCGAGAAGATGA | CCAGAGGCGTACAGGGATAG | 138 |
| *GAPDH* | NM_001289746.1 | CGACCACTTTGTCAAGCTCA | GGGTCTTACTCCTTGGAGGC | 206 |
| *NEUROG3* | NM_020999.3 | AGCCGGCCTAAGAGCGAGTT | TTGGTGAGCTTCGCGTCGTC | 158 |

### Supplementary Table 2. qRT-PCR primers

Forward and reverse primer sequences are given for each gene along with accession number and product size. All primer pairs / products were intron-spanning wherever possible.
